# Supplementary material for: Effects of Morphology and Solvent/Temperature on THz Spectra: Take Nucleosides as Example
Source: Molecules. 2023 Feb 4;28(4):1528. doi: 10.3390/molecules28041528 (PMC9965851; doi:10.3390/molecules28041528)
Supplement: Supplementary file 1 [file molecules-28-01528-s001.zip › molecules-2169109-supplementary.pdf]

Effects of morphology and solvent / temperature on THz spectra: take nucleosides as example

Fang Wang[1], Haifeng Lin[2*], Jiawen Tong[1], Junbin Tai[1], Jiaen Wu[1], Yaru Yao[1], Yunfei Liu[2]

[1]College of Electronic Engineering, Nanjing XiaoZhuang University, Nanjing 211171, China
[2]College of Information Science and Technology, Nanjing Forestry University, Nanjing 210037, China
*Correspondence: haifeng.lin@njfu.edu.cn

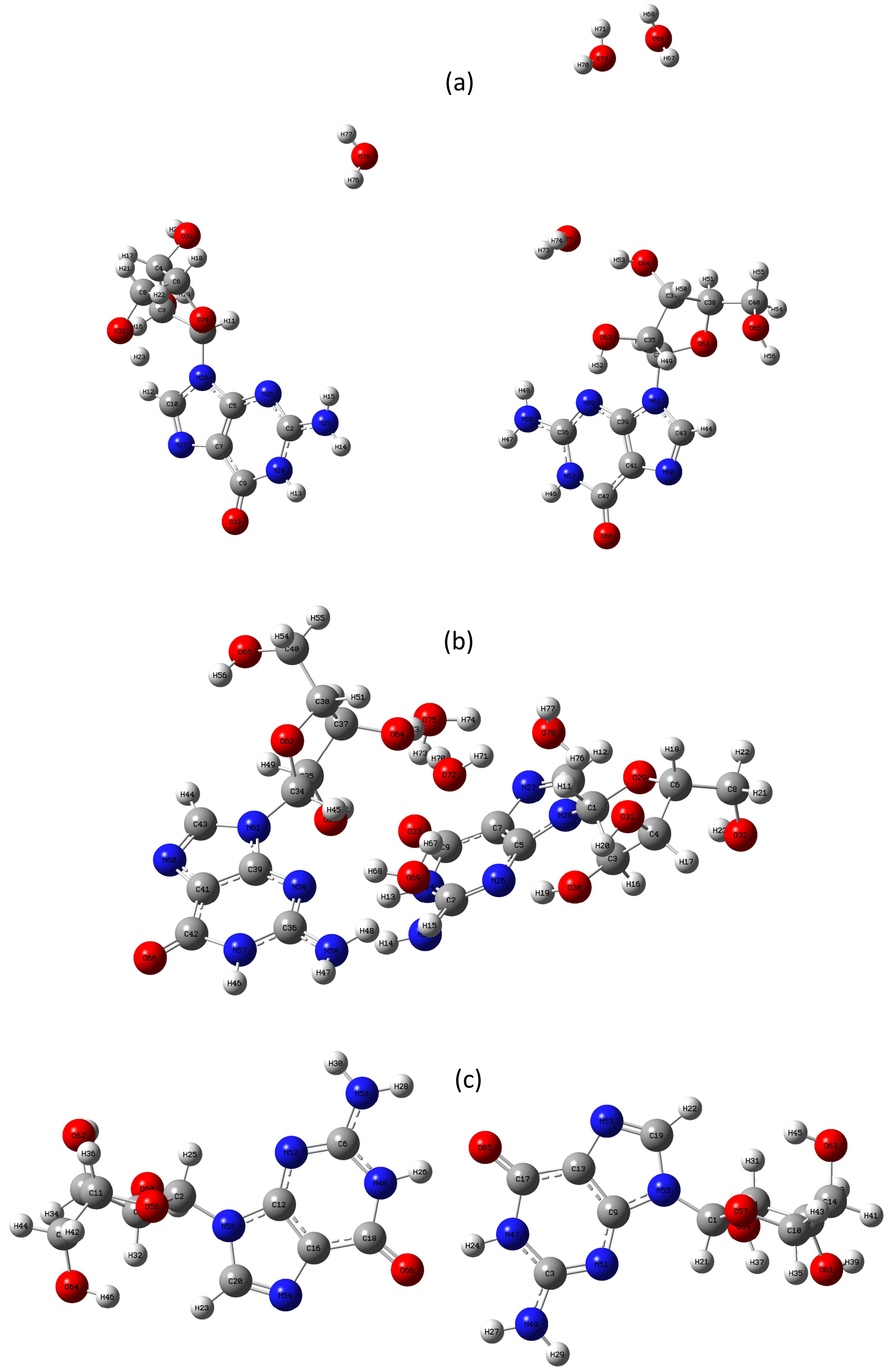

(a)

(b)

(c)

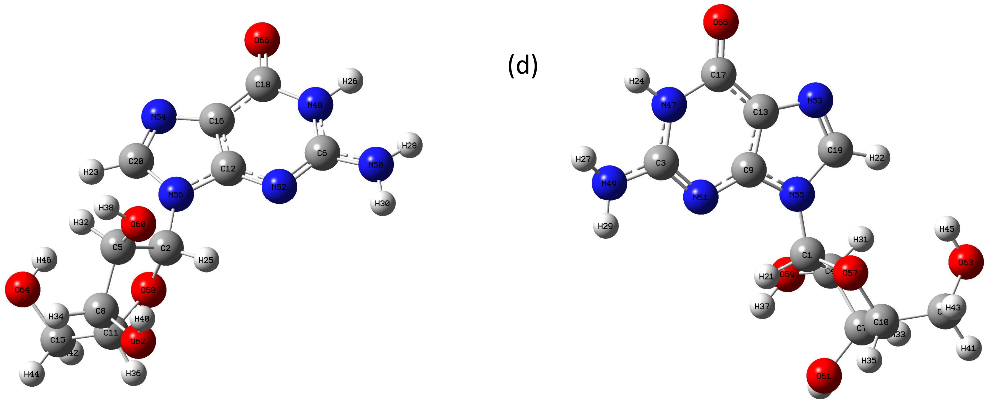

Figure S1. The structure of (a) Guanosine-dimer-2H2O before optimization, (b) and the structure of Guanosine-dimer-2H2O, (c) Guanosine-dimer, (d) Guanosine-dimer-water after optimization.

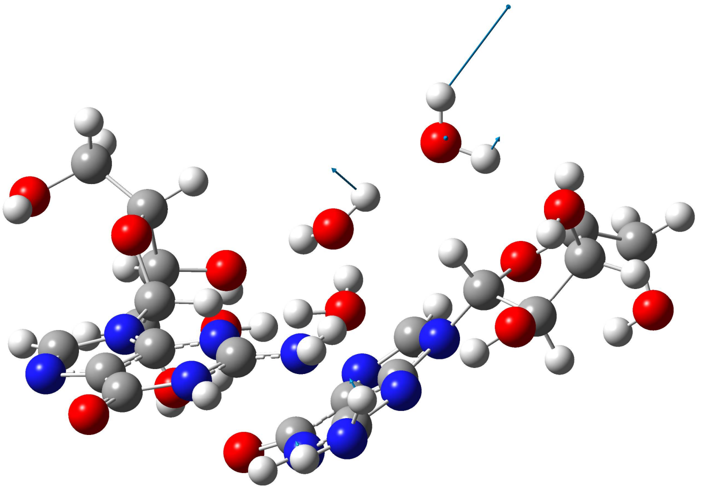

5.7 THz (Guanosine-dimer-2H2O)

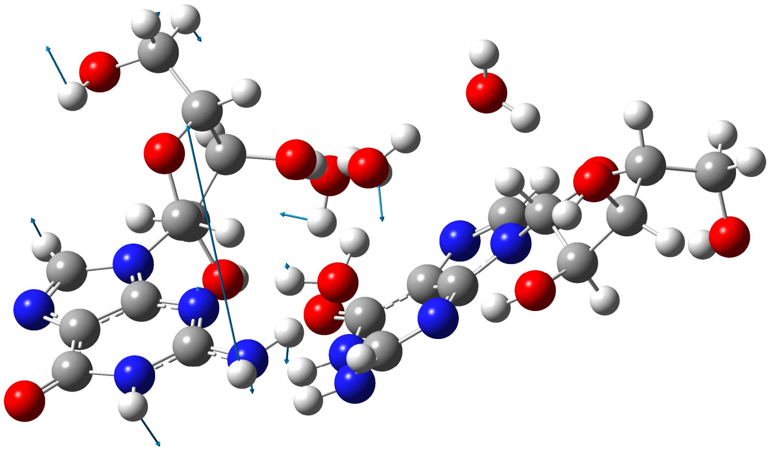

9.7 THz (Guanosine-dimer-2H2O)

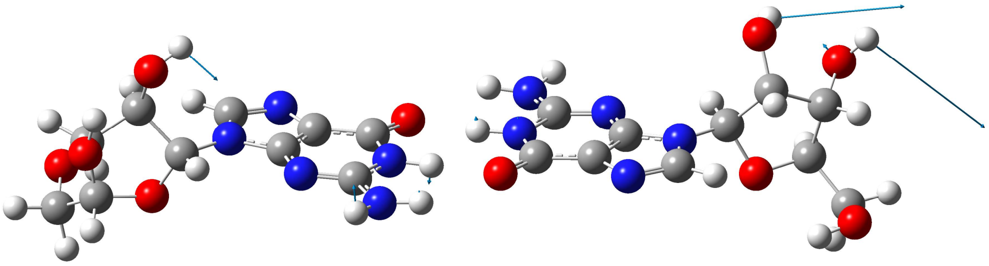

5.5 THz (Guanosine-dimer)

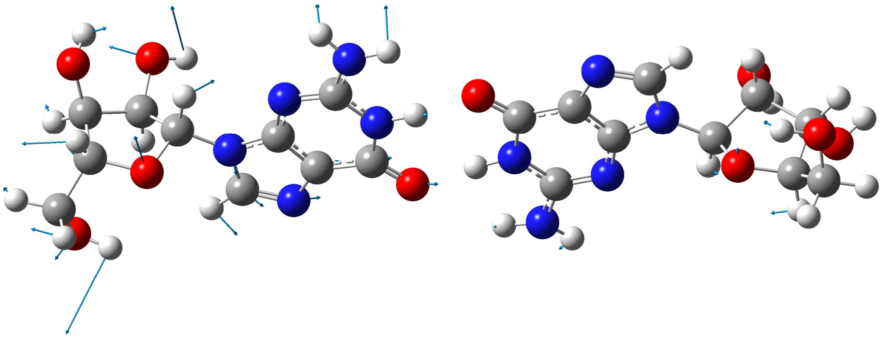

7.1 THz (Guanosine-dimer)

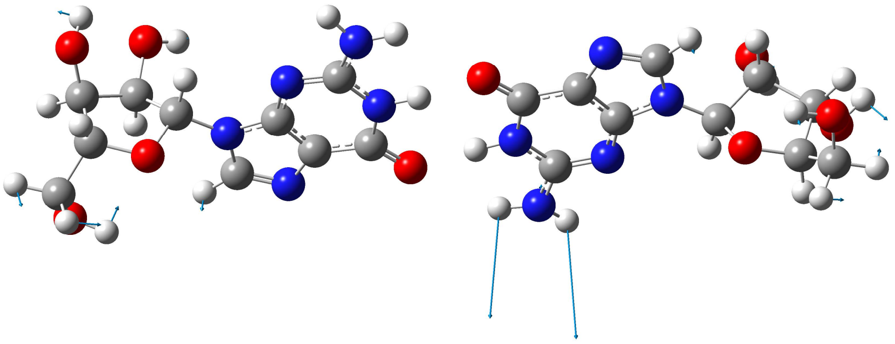

8.7 THz (Guanosine-dimer)

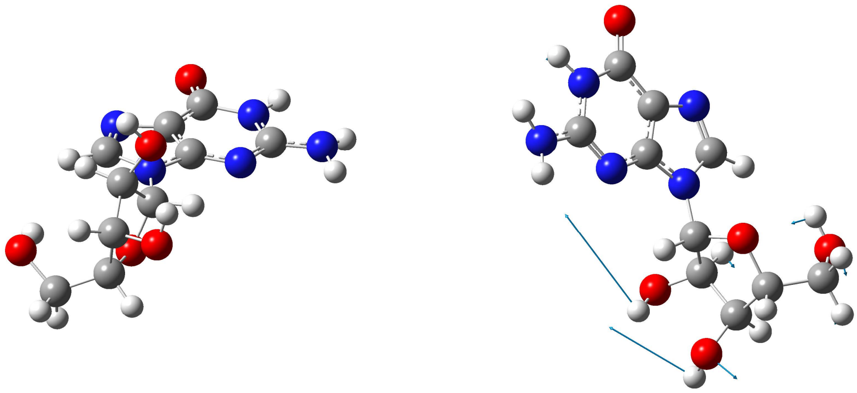

6.2 THz (Guanosine-dimer-water)

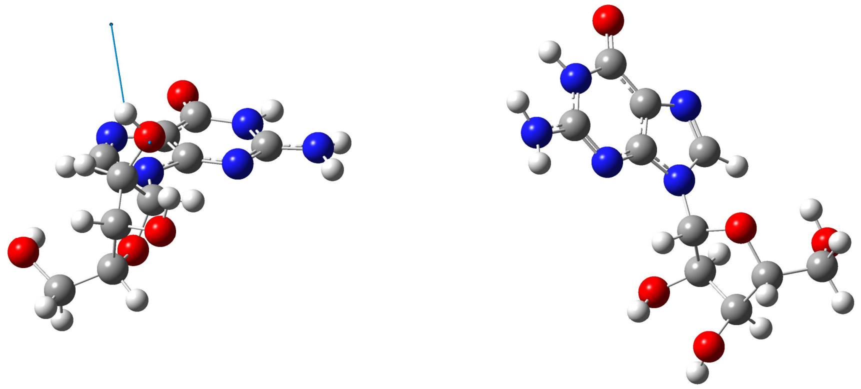

7.2 THz (Guanosine-dimer-water)

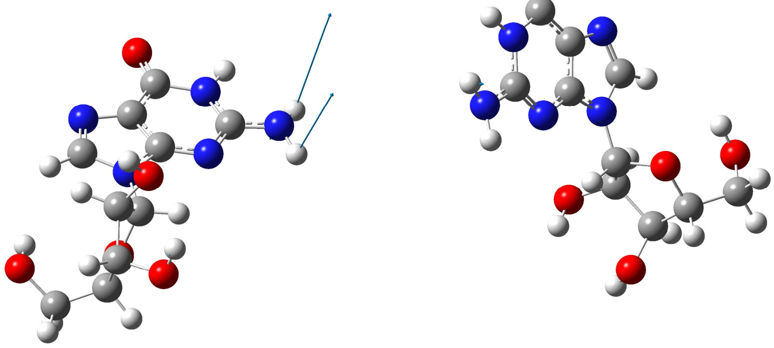

9.5 THz (Guanosine-dimer-water)

Figure S2. The vibrational modes corresponding to the main characteristic peaks of Guanosine-dimer-2H2O, Guanosine-dimer and Guanosine-dimer-water.
